# microRNA function in left-right neuronal asymmetry: perspectives from C. elegans

**DOI:** 10.3389/fncel.2013.00158

**Published:** 2013-09-23

**Authors:** Amel Alqadah, Yi-Wen Hsieh, Chiou-Fen Chuang

**Affiliations:** ^1^Division of Developmental Biology, Cincinnati Children’s Hospital Research FoundationCincinnati, OH, USA; ^2^Molecular and Developmental Biology Graduate Program, University of CincinnatiCincinnati, OH, USA

**Keywords:** microRNA, neuronal asymmetry, sensory neurons, calcium signaling, *C. elegans*

## Abstract

Left–right asymmetry in anatomical structures and functions of the nervous system is present throughout the animal kingdom. For example, language centers are localized in the left side of the human brain, while spatial recognition functions are found in the right hemisphere in the majority of the population. Disruption of asymmetry in the nervous system is correlated with neurological disorders. Although anatomical and functional asymmetries are observed in mammalian nervous systems, it has been a challenge to identify the molecular basis of these asymmetries. *C. elegans* has emerged as a prime model organism to investigate molecular asymmetries in the nervous system, as it has been shown to display functional asymmetries clearly correlated to asymmetric distribution and regulation of biologically relevant molecules. Small non-coding RNAs have been recently implicated in various aspects of neural development. Here, we review cases in which microRNAs are crucial for establishing left–right asymmetries in the *C. elegans* nervous system. These studies may provide insight into how molecular and functional asymmetries are established in the human brain.

## INTRODUCTION

microRNAs (miRNAs) are endogenous 20–24 nt small non-coding RNAs that regulate gene expression through binding to complementary sequences in target messenger RNAs (mRNAs), leading to translational repression and/or cleavage of target mRNAs (Ambros, 2004; He and Hannon, 2004; Bartel, 2009; Chekulaeva and Filipowicz, 2009; Ghildiyal and Zamore, 2009). While most miRNAs downregulate gene expression, there are examples of miRNA-mediated upregulation of target gene expression during cell cycle arrest, suggesting that miRNA function is complex and context dependent (Vasudevan et al., 2007; Orom et al., 2008). miRNAs have been implicated in many aspects of development and disease including cell cycle, cell differentiation, apoptosis, life span, developmental timing, stress responses, neural development and regeneration, cancers, and neurodegenerative disorders (Boehm and Slack, 2005; Bushati and Cohen, 2007; Chang et al., 2009; Ambros, 2011; Sayed and Abdellatif, 2011; Zhang et al., 2011; Boulias and Horvitz, 2012; Cochella and Hobert, 2012a; Saito and Saito, 2012; Zou et al., 2012, 2013).

## FUNCTIONS OF miRNAs IN NEURAL DEVELOPMENT

The importance of miRNAs in various aspects of neuronal development has been demonstrated in several animal models. In zebrafish, maternal–zygotic *dicer* mutants, that disrupt the processing of precursor miRNAs into mature miRNAs, display deleterious effects on the development of the brain, and injection of mature miR-430 rescues the early brain patterning defects (Giraldez et al., 2005). In mice, the neuron-specific miRNA miR-124 induces neuronal differentiation by directly targeting a global repressor of alternative pre-mRNA splicing and triggering a downstream switch to neuron-specific alternative splicing (Makeyev et al., 2007). In the *Xenopus* retina, a number of cell cycle related miRNAs target Xotx2 and Xvsx1 in early retinal progenitor cells to inhibit bipolar cell differentiation (Decembrini et al., 2009). In *Drosophila*, miR-9a targets senseless to inhibit neuronal fate in non-sensory organ precursors (Li et al., 2006). In *C. elegans*, the miRNA *lin-4* targets the LIN-14 transcription factor to inhibit netrin-mediated axon attraction (Chang et al., 2004a; Zou et al., 2012), and the miRNA *let-7* contributes to a developmental decline in neuronal regeneration (Zou et al., 2013). In addition, miRNAs *lsy-6*, *mir-273*, and *mir-71* function in asymmetric differentiation of two pairs of *C. elegans* sensory neurons, which will be discussed later (Johnston and Hobert, 2003; Chang et al., 2004b; Hsieh et al., 2012). Thus, miRNAs are important factors that control neuronal development across the animal kingdom.

## LEFT–RIGHT ASYMMETRY OF THE NERVOUS SYSTEM

Although the nervous systems of animals are largely symmetric across the left–right axis, there have been several observations of anatomical and functional brain lateralization throughout the animal kingdom. For example, zebrafish display asymmetry in the epithalamus (Snelson and Gamse, 2009; Taylor et al., 2010); mice have been shown to have paw preferences, indicating the presence of a dominant hemisphere in motor control (Signore et al., 1991; Biddle et al., 1993); and the majority of humans have language centers such as Wernicke’s and Broca’s area located in the left hemisphere of the brain (Sun and Walsh, 2006). This lateralization of the nervous system is thought to be beneficial, as it allows for an increase of functional capacity (Rogers et al., 2012).

Disruption of asymmetry in the brain is seen in a number of neurodevelopmental diseases, including dyslexia, schizophrenia, autism, Alzheimer’s disease, and attention deficit/hyperactivity disorder (ADHD; Herbert et al., 2005; Derflinger et al., 2011; Oertel-Knochel and Linden, 2011; Renteria, 2012). In children diagnosed with ADHD, the prefrontal cortex was shown to have a loss in rightward asymmetric distribution of prefrontal cortex volume as compared to typically developing children (Shaw et al., 2009). In dyslexic patients, the planum temporale shows alteration in asymmetry, as the right temporale appears physically larger than the left counterpart (Galaburda et al., 1985).

There have been several reports on functional and anatomical nervous system asymmetries; however there have been comparatively fewer studies on identifying the molecular mechanisms that establish lateralization. Here we narrow our focus to the function and regulation of miRNAs in the development of neuronal asymmetry. The most interesting evidence of miRNAs in vertebrate neuronal asymmetry comes from the investigation of miRNA function in neocortex development. The simplest form of asymmetry is the division of a cell to give rise to two asymmetric fates. In the case of the mouse neocortex, a progenitor cell divides asymmetrically to give rise to a progenitor cell and a neuron. Asymmetric localization of the TRIM-NHL protein TRIM32 is observed in the daughter cell that becomes a neuron, while the cell lacking TRIM32 remains a progenitor (Schwamborn et al., 2009). TRIM32 increases the activity of specific miRNAs through binding of the RNAse argonaute-1 (Hammell et al., 2009; Schwamborn et al., 2009). It was further shown that the miRNA Let-7a, one of the TRIM32 targets, is required and sufficient for neuronal differentiation (Schwamborn et al., 2009).

A subset of zebrafish olfactory bulb output neurons called the mitral cells send axons asymmetrically to the right habenula, which is an asymmetric part of the brain where the higher olfactory processing center is located (Miyasaka et al., 2009; Taylor et al., 2010). Several molecules are asymmetrically expressed in adult-born olfactory neurons in the ventricular–subventricular zone (V–SVZ) of the zebrafish brain. The transcription factors Myt1 and Neurogenin1 are predominantly expressed in the left V–SVZ, while DeltaA and hairy/enhancer of split-related protein (a Notch effector) are mainly expressed on the right side (Kishimoto et al., 2013). In addition, members of the miR-200 family are involved in the proper differentiation of olfactory neurons of both mice and zebrafish (Choi et al., 2008). It would be interesting to see whether the miR-200 family or other miRNAs influences the asymmetric and lateral projection of olfactory axons to the higher olfactory centers, and whether Myt1, Neurogenin1, and Delta/Notch are potential targets of miR-200.

A study found that 27 genes are differentially expressed in the embryonic human cerebral cortex, and the Lim domain transcription factor LMO4 is more abundant in the right perisylvian cortex than the left, and may be involved in asymmetric development of the cortex (Sun et al., 2005). The hypothesis that differential expression of these genes between left and right sides of the cortex may be regulated by miRNAs is plausible and worth further investigation.

Although there are limited reports of the involvement of miRNAs in the development of neuronal asymmetry in vertebrates, *C. elegans* has proved to be a powerful model organism to study lateralization of the nervous system due to its genetic amenability, as well as evidence of functional asymmetry having clear molecular correlates. In this review, we highlight two cases of the roles that miRNAs play in establishing left/right asymmetry in the *C. elegans* nervous system. Both involve the specification of two types of chemosensory neurons: the pair of amphid neurons, single cilliated endings (ASE) taste neurons, in which *lsy-6* and *mir-273* miRNAs are involved, and the pair of amphid wing “C” (AWC) olfactory neurons, where *mir-71* is crucial for establishment of asymmetry.

## miRNAs IN TASTE NEURON ASYMMETRY

Like other animals, the *C. elegans* nervous system appears generally symmetric. However, the pair of taste neurons, called ASE left (ASEL) and ASE right (ASER) displays molecular and functional asymmetries. The ASE neurons are located in the nerve ring of the nematode, which is the brain equivalent in the worm. Although the neurons are derived from different cell lineages, they are anatomically symmetric in terms of cell position, morphologies, and axonal projections (White et al., 1986). The ASEL neuron, however, differentially expresses the putative chemoreceptor *gcy-7*, while the ASER neuron expresses another putative chemoreceptor *gcy-5* (Yu et al., 1997). The pair of ASE neurons also senses different chemicals, as the ASEL neuron functions to detect sodium, while ASER senses chloride (Pierce-Shimomura et al., 2001).

**FIGURE 1:**
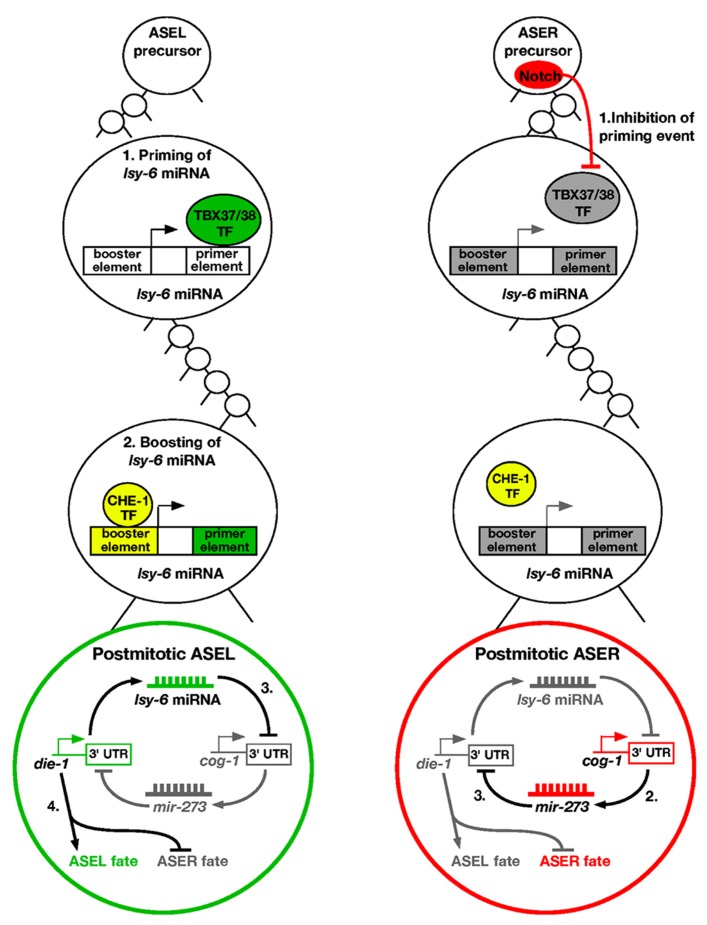
**Priming and boosting model of ASE asymmetry.** ASEL: (1) The first step of promoting the ASEL cell fate occurs six cell divisions before the postmitotic ASEL is born. A transient pair of T-box transcription factors TBX-37/38 acts on the downstream primer element of *lsy-6* miRNA to open up the chromatin. (2) The priming event allows boosting of *lsy-6* miRNA levels by the CHE-1 transcription factor in the ASEL mother cell, leading to high levels of the miRNA in the postmitotic ASEL neuron. (3) The *lsy-6* miRNA directly inhibits the ASER promoting transcription factor COG-1, and allows the ASEL promoting transcription factor DIE-1 to be expressed. (4) DIE-1 then activates ASEL effector genes and represses the ASER cell fate. ASER: (1) Notch is expressed in the ASER precursor and inhibits the *lsy-6* priming event. Therefore, the *lsy-6* miRNA expression level cannot be boosted as the chromatin remains compact. (2) Consequently, in the postmitotic ASER neuron, the COG-1 transcription factor is expressed, which activates another miRNA, *mir-273*. (3) *mir-273* directly inhibits the ASEL promoting transcription factor DIE-1, and therefore promotes the ASER fate.

Over the past few years, there have been many studies investigating the molecular mechanism on how ASE asymmetry is established. Intriguingly, the first step of breaking symmetry of the taste neurons occurs several divisions before the ASEL and ASER neurons are born, during the early embryonic stage (Poole and Hobert, 2006). In the ASEL lineage, a pair of redundant T-box transcription factors TBX-37/38 are transiently expressed six cell divisions before the birth of ASEL (Good et al., 2004; **Figure 1**). These transcription factors work to “prime” a miRNA called *lsy-6*, which promotes the ASEL cell fate. This is achieved by binding of TBX-37/38 to a downstream primer element of *lsy-6* and results in physical opening up of the *lsy-6* chromatin (Cochella and Hobert, 2012b). The priming event is then “remembered” several cell divisions later in the ASEL mother cell. The open chromatin status of *lsy-6* allows for the CHE-1 zinc finger transcription factor to bind to an upstream booster element of the *lsy-6* locus (Cochella and Hobert, 2012b). This induces “boosting” of *lsy-6* expression levels in the ASEL neuron. The “prime and boost” model is essential for establishing ASE asymmetry.

In the ASER lineage, a Delta/Notch signal in the ASER precursor cell causes the T-box transcription factors TBX-37/38 to be repressed (Good et al., 2004; Priess, 2005). Therefore, the *lsy-6* “priming” event does not occur in the ASER lineage, allowing the *lsy-6* chromatin to remain in a compact form (Cochella and Hobert, 2012b). This in turn leads to the inability of the CHE-1 transcription factor to physically bind the upstream element of *lsy-6*, and no boosting of the miRNA expression levels occurs in ASER. Overall, this causes asymmetric distribution of the *lsy-6* miRNA in the ASEL neuron.

The *lsy-6* miRNA functions in a double negative feedback loop to control the asymmetry of ASEL/R neurons (Johnston et al., 2005). In the ASEL neuron, *lsy-6* directly represses an ASER promoting transcription factor COG-1, through physical binding of complementary bases in the *cog-1* 3′UTR (Johnston and Hobert, 2003). Repression of COG-1 allows for yet another transcription factor DIE-1 to be expressed, which is the output of the feedback loop. DIE-1 then activates ASEL effector genes and suppresses ASER effector genes (Johnston et al., 2005; **Figure 1**).

In the ASER neuron, *lsy-6* expression is relatively low, and cannot effectively repress the COG-1 transcription factor. This allows activation of another miRNA, *mir-273*, which displays complementarity to the 3′UTR of the previously described *die-1* output (Chang et al., 2004b). *mir-273* therefore represses DIE-1, resulting in the de-repression of the ASER fate (**Figure 1**). Mutating any of the factors involved results in a loss of asymmetry, with both cells adopting either the ASEL or ASER cell fate.

## miRNAs IN OLFACTORY NEURON ASYMMETRY

**FIGURE 2:**
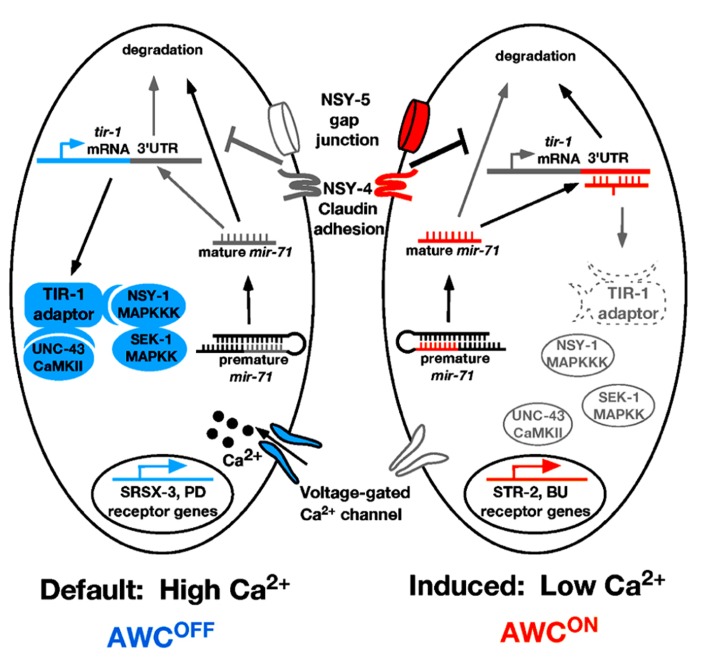
**Model of *mir-71* function in AWC asymmetry.** In the AWC^OFF^ cell, high calcium level activates the calcium-regulated UNC-43/TIR-1/MAPK cascade, leading to *srsx-3* expression. In the AWC^ON^ cell, *nsy-4* and *nsy-5* promote the stability of mature *mir-71* through an unknown mechanism. The mature *mir-71* miRNA targets the 3′UTR of *tir-1* mRNA for degradation, leading to the inhibition of the calcium signaling pathway and the subsequent induction of *str-2* expression. Orange color represents factors that promote AWC^ON^. Blue color represents factors that promote AWC^OFF^. Gray/white color represents inactive or less active factors. Dotted line represents factors being downregulated. PD, pentanedione; BU, butanone.

Like the ASE neurons, the *C. elegans* AWC olfactory neurons are bilaterally symmetrical at the morphological level. However, AWC left (AWCL) and AWC right (AWCR) neurons express different odorant receptors and sense different odors (**Figure 2**; Troemel et al., 1999). The AWC^ON^ neuron expresses the odorant receptor gene *str-2* and specifically senses the odor butanone; while the contralateral AWC^OFF^ neuron expresses the odorant receptor gene *srsx-3* and specifically senses the odor pentanedione (**Figure 2**; Troemel et al., 1999; Wes and Bargmann, 2001; Bauer Huang et al., 2007). In wild-type animals, only one of the AWC neurons expresses *str-2*. AWC asymmetry is stochastic and coordinated, so that 50% of the worms in a population express *str-2* in AWCL, while the other 50% express *str-2* in AWCR (Troemel et al., 1999; Taylor et al., 2010). The default state of the AWC neurons is AWC^OFF^, which is specified by a calcium-regulated and microtubule-dependent MAP kinase pathway including UNC-43/CaMKII, TIR-1/Sarm1, NSY-1/MAPKKK, and SEK-1/MAPKK (**Figure 2**; Sagasti et al., 2001; Tanaka-Hino et al., 2002; Chuang and Bargmann, 2005; Chang et al., 2011). NSY-4 claudin-like protein and NSY-5 gap junction protein act in parallel to inhibit the calcium signaling pathway in the induced AWC^ON^ cell (Vanhoven et al., 2006; Chuang et al., 2007). In addition, intercellular calcium signaling between AWCs and non-AWC neurons via a NSY-5 gap junction-dependent neural network coordinates precise AWC asymmetry (Schumacher et al., 2012).

One of the important questions on AWC asymmetry is how the calcium signaling pathway is downregulated by *nsy-4* and *nsy-5* in the AWC^ON^ cell. A recent study showed that the miRNA *mir-71* acts downstream of *nsy-4* and *nsy-5* to promote AWC^ON^ in a cell autonomous manner through inhibiting the expression of the calcium signaling adaptor protein gene *tir-1* (**Figure 2**; Hsieh et al., 2012). The TIR-1/Sarm1 adaptor protein assembles a calcium-signaling complex to cell-autonomously specify the default AWC^OFF^ identity (Chuang and Bargmann, 2005). Thus downregulation of *tir-1* expression by *mir-71* is an efficient mechanism to inhibit calcium signaling in the cell becoming AWC^ON^.

*mir-71* is regulated at both transcriptional and post-transcriptional levels in AWC (Hsieh et al., 2012). At the transcriptional level, the expression level of *mir-71* is higher in the AWC^ON^ cell than in the AWC^OFF^ cell. This transcriptional bias of *mir-71* is not dependent on *nsy-4* or *nsy-5*; thus, the mechanisms that regulate differential expression of *mir-71* in the two AWC cells are yet to be elucidated. At the post-transcriptional level, the stability of mature *mir-71* is dependent on *nsy-4* and *nsy-5*. The *C. elegans* 5′ → 3′ exoribonuclease XRN-2 has been shown to be involved in degradation of mature miRNAs (Chatterjee and Grosshans, 2009). It is possible that *nsy-4* and *nsy-5* may antagonize the *xrn-2*-mediated miRNA turnover pathway to increase the level of mature *mir-71*. However, RNA interference (RNAi) knockdown of *xrn-2* did not cause a defect in AWC asymmetry, suggesting that the stability of mature *mir-71* may be independent of *xrn-2*. In support of this idea, not all miRNAs accumulate in *xrn-2* RNAi worms (Chatterjee and Grosshans, 2009), suggesting the existence of alternative miRNA turnover pathways that may be inhibited by *nsy-4* and *nsy-5*.

## PERSPECTIVES

Understanding the molecular mechanisms involved in establishing left–right asymmetry in the *C. elegans* nervous system can lay the groundwork for identifying the processes used in higher organisms, as the methods used may be evolutionarily conserved. Because of the highly conserved nature of miRNAs, insights into how they are involved to control asymmetric fates will help facilitate our understanding in vertebrate neuronal asymmetry. The involvement of miRNAs in asymmetry may also be reflective of the principles these small non-coding RNAs use in directing other neurodevelopmental processes.

## Conflict of Interest Statement

The authors declare that the research was conducted in the absence of any commercial or financial relationships that could be construed as a potential conflict of interest.
